# 

**Published:** 1883-04

**Authors:** 


					﻿ESTABLISHED IN 1B55.
Old Serie®.	APPTT IGfiQ	New Setihs.
Vol. XXI-No.J^Z^_1OOO.	Vol. Il—No. 7.
----,
vi)
ATLANTA
MEDICAL REGISTER.
{ATLANTA MEDICAL AND SURGICAL JOURNAL.)
JAMES B. BAIRD, M.D.
e
H.H. DICKSON. PUBLISHER. TERMS, $2-50 A YEAR IN ADVANCE
ATLANTA, GEORGIA:
II. II. DICESQB', BOOK ABD JOB PR1BTER ABD B00EB1BDER
1883.
				

